# Mitotic Chromosomes in Live Cells Characterized Using High-Speed and Label-Free Optical Diffraction Tomography

**DOI:** 10.3390/cells8111368

**Published:** 2019-10-31

**Authors:** Tae-Keun Kim, Byong-Wook Lee, Fumihiko Fujii, Kee-Hang Lee, Sanghwa Lee, YongKeun Park, Jun Ki Kim, Sang-Wook Lee, Chan-Gi Pack

**Affiliations:** 1 Asan Institute for Life Sciences, Asan Medical Center, Seoul 05505, Korea; m996142@gmail.com (T.-K.K.); sorrow98@nate.com (B.-W.L.); pause1919@gmail.com (S.L.); 2 Division of Physical Pharmacy, Faculty of Pharmaceutical Sciences, Kobe Gakuin University, Kobe 650-8586, Japan; ffujii4@gmail.com; 3 Tomocube Inc., Daejeon 34051, Korea; khlee@tomocube.com (K.-H.L.);; 4 Department of Physics, Korea Advanced Institute of Science and Technology, Daejeon 34141, Korea; 5 Department of Convergence Medicine, University of Ulsan College of Medicine, Seoul 05505, Korea; 6 Department of Radiation Oncology, University of Ulsan College of Medicine, Asan Medical Center, Seoul 05505, Korea

**Keywords:** optical diffraction tomography, fluorescence correlation spectroscopy, mitosis, chromosome, refractive index, cellular viscosity, diffusion coefficient, osmotic stress

## Abstract

The cell nucleus is a three-dimensional, dynamic organelle organized into subnuclear compartments such as chromatin and nucleoli. The structure and function of these compartments are maintained by diffusion and interactions between related factors as well as by dynamic and structural changes. Recent studies using fluorescent microscopic techniques suggest that protein factors can access and are freely mobile in heterochromatin and in mitotic chromosomes, despite their densely packed structure. However, the physicochemical properties of the chromosome during cell division are not fully understood. In the present study, characteristic properties such as the refractive index (RI), volume of the mitotic chromosomes, and diffusion coefficient (*D*) of fluorescent probes inside the chromosome were quantified using an approach combining label-free optical diffraction tomography with complementary confocal laser-scanning microscopy and fluorescence correlation spectroscopy. Variations in these parameters correlated with osmotic conditions, suggesting that changes in RI are consistent with those of the diffusion coefficient for mitotic chromosomes and cytosol. Serial RI tomography images of chromosomes in live cells during mitosis were compared with three-dimensional confocal micrographs to demonstrate that compaction and decompaction of chromosomes induced by osmotic change were characterized by linked changes in chromosome RI, volume, and the mobilities of fluorescent proteins.

## 1. Introduction

The cell nucleus is dynamically and three-dimensionally organized into subnuclear compartments including chromatin, chromosomes, and nuclear bodies such as nuclear speckles, Cajal bodies, and nucleoli comprising a high density of macromolecules. In particular, genomic DNA is organized into chromatin in the nucleus to make genomic information accessible for physiological function. It is unclear how protein factors find and interact with their targets, and the structure and function of nuclear compartments are likely maintained by mobility and interaction with related factors during the cell cycle. Studies using biophysical methods such as fluorescence correlation spectroscopy (FCS) demonstrate that molecular probes freely diffuse in the nucleus and subnuclear compartments such as chromatin, chromosomes, and nucleoli [1,2,3,4]. The cellular microenvironment and molecular crowding are key physicochemical characteristics of cellular compartments with a high macromolecular content [5,6,7,8,9]. Therefore, diffusion coefficients (*D*s) of fluorescent probes with well-characterized structures and molecular weights, such as green fluorescent protein (GFP) in live cells have been systematically characterized to investigate these characteristics [3,4,10,11,12,13,14]. In particular, observations using FCS suggest that heterochromatin and mitotic chromosomes are accessible to protein factors with molecular weights up to 150 kDa (i.e., pentameric GFP probes), despite their densely packed structure [3]. Furthermore, nucleosomes as well as probe proteins may be freely mobile in these compartments. However, the physicochemical properties of mitotic chromosomes under various physiological conditions are not fully understood.

Previous studies have focused only on the diffusional mobility of probes but not on the molecular densities of target compartments. The *D* values of probe molecules within nuclear compartments such as the nucleolus and mitotic chromosomes do not provide the absolute molecular density of the compartment but the fluidic property of surrounding microenvironment [3,4]. It is predicted that the *D* value of a probe molecule is not necessarily proportional to molecular density, because the mobility of the surrounding molecules affects the mobility of the probe, and the degree of freedom can be changed by physiological conditions.

FCS based on confocal laser-scanning microscopy (CLSM) is a highly sensitive technique for quantitatively assessing molecular concentrations and diffusion of fluorescent probes in aqueous solutions and living cells [1,15,16,17]. FCS is highly sensitive and requires only a small detection volume (~0.15 fl). Therefore, it is well-suited to measuring the diffusion of probe molecules in very small regions that comprise subnuclear compartments in living cells. However, FCS measurements of large areas are time-consuming and too inefficient to allow simultaneous volumetric measurement of slowly mobile compartment such as the mitotic chromosome. Moreover, the phototoxic and bleaching effects of fluorescence methods such as confocal microscopy and FCS must be also carefully considered when attempting to obtain reliable information from live cells, especially mitotic cells. To overcome the disadvantages of fluorescence methods, we used three complementary methods in a single approach, combining the label-free quantitative phase-imaging (QPI) method with CLSM and confocal-based FCS. The label-free and fast QPI method may compensate for the limitations of the two fluorescence methods, such as phototoxicity originating from fluorescent labels, long scanning times for three-dimensional (3D) imaging of CLSM, and time-consuming multi-point measurements of FCS. Recently, a label-free QPI method such as optical diffraction tomography (ODT) was identified as a promising method for high-speed live cell imaging capable of compensating for the limitations of fluorescent imaging [18,19,20,21,22,23], even though the quality of 3D images of cellular organelles has not yet been fully compared between CLSM and ODT.

Furthermore, because low light intensities are required for object illumination, ODT minimizes photostress on the transparent biological sample, making it suitable for the non-invasive measurement of live cells during mitosis. In addition to imaging live cells, ODT simultaneously provides analytical information on absolute biophysical parameters such as the volume of cells and the refractive index (RI) [24]. The RI is generally proportional to the concentration of organic solutes (i.e., molecular density) which, in turn, is related to the viscosity of aqueous solutions [25]. Therefore, correlation methods such as FCS, image correlation spectroscopy, and ODT may be complementary. A previous study demonstrated that label-free phase correlation imaging (PCI) based on QPI simultaneously provides two biophysical parameters for analyses of cell dynamics: the diffusion coefficient of mass transport (~0.1 μm^2^/s) and the RI [26]. However, PCI is limited in that it provides no information about the fluidic viscosity of each cellular compartment. In contrast, FCS based on CLSM is useful for detecting a broad range of diffusion rates (0.1–100 μm^2^/s) of fluorescent probes inside a dynamic and compact structure. Optical diffraction tomography is an interferometric microcopy technique that acquires 3D and time-lapse RI tomograms of cells (i.e., 4D imaging) and tissues without prior preparation or labeling. Therefore, ODT microscopy can observe unfixed cells and unlabeled, living cells without fluorescent protein expression or immunofluorescence. Moreover, ODT imaging is much faster than CLMS imaging and can acquire one 3D RI tomogram in <1 s [27].

Male Indian Muntjac (DM) cells have 2n = 7 diploid chromosomes that are large compared to those of common cell lines such as HeLa. Therefore, the DM cell line is ideal for visualizing mitotic chromosomes using the H2B marker protein tagged with monomeric red fluorescent protein (H2B-mRFP) and for measuring the diffusion of fluorescent probe proteins through the chromosome. In a previous study, DM cells co-expressing H2B-mRFP and probe green fluorescent protein (GFP) were subjected to fluorescence imaging methods such as CLSM imaging and FCS analyses [3]. In the present study, DM cells co-expressing H2B-mRFP and monomeric GFP (mGFP) are exploited to enable a direct comparison between ODT, fluorescent confocal 3D micrography, and FCS. Monomeric GFP was used as a fluorescent probe to quantify the diffusion coefficient (*D)* and local viscosity in the mitotic chromosome.

We demonstrate the application of our method for quantification of physical parameters of the mitotic chromosome, including volume, RI, and *D* for fluorescent probes inside the chromosome. We acquired 3D-RI images of mitotic chromosomes of DM cells and combined these analyses with those using CLSM and FCS. The tomographic RI images of DM cells with a small number of large chromosomes showed a clear structure of chromatids compared with that of HeLa cells. The RI tomographic images DM chromosomes were consistent with the fluorescence images of these cells expressing H2B-mRFP. Moreover, we compared the RI values of chromosomes quantitatively, molecular accessibilities, and *D* values of the mGFP probe inside the mitotic chromosome under various osmotic conditions. Confocal and RI images showed that chromosomes in the mitotic cells were significantly compacted when culture medium was replaced with a solution of high osmolality, indicating that hypotonic stress induced decompaction of the chromosomes. This chromosomal compaction and decompaction was characterized by a change in RI, consistent with the changes of *D* values of mGFP probes.

## 2. Materials and Methods

### 2.1. Cells

DM cells stably expressing H2B-mRFP and mGFP [1,3] were kindly provided by Dr. Maeshima (National Institute of Genetics, Mishima, Japan). DM cells were cultured at 37 °C in an atmosphere containing 5% CO_2_ in Dulbecco’s modified Eagle’s medium (DMEM) supplemented with 20% fetal bovine serum (FBS), 100 U/mL penicillin, and 100 U/mL streptomycin. HeLa cells were cultured at 37 °C in an atmosphere containing 5% CO_2_ in DMEM supplemented with 10% FBS, 100 U/mL penicillin, and 100 U/mL streptomycin. For live cell microscopy, stably-transfected DM cells were plated in Tomo-dishes (Tomocube, Daejeon, Korea) or Lab-Tek 8-well chambered coverglasses (Nunc, Rochester, NY, USA). To observe HeLa cells expressing H2B-mRFP, cells were transiently transfected with the H2B vector using Lipofectamine 3000 (Thermo Fisher Scientific, Waltham, MA, USA).

To investigate the influence of osmotic stress, DM cells, which are normally cultured and maintained in a DMEM with high osmolarity (i.e., refractive index), containing 20% FBS, were incubated with phosphate-buffered saline (PBS) (hypoosmolar) or DMEM containing 0.2 M sucrose and 20% FBS (hyperosmolar) (Table 1). Media were warmed to 37 °C before use. For HeLa cells, PBS or phenol-free DMEM supplemented with 10% FBS and 0.2 M sucrose was added before imaging. Dulbecco’s PBS (Biowest, Nuaille, France) was used to wash DM and HeLa cells.

### 2.2. Osmotic Concentration, Refractive Index (RI), and Culture Media

The osmotic concentrations of media were measured using an osmometer (Fiske Micro-Osmometer Model 210, Advanced Instruments, MA, USA). The RIs of glycerol–water solutions and culture media were measured using a refractometer at 15 °C to 60 °C (Abbemat 550; Anton Paar GmbH, Graz, Austria) (Table 1). Glycerol solutions, purity >99.5% (Biosesang, Seongnam, South Korea) and 10%, 20%, 30%, 40%, 50%, 60%, and 70% (*w*/*v*) were used to determine the relationship between viscosity and RI.

### 2.3. Optical Diffraction Tomography (ODT)

ODT was performed as described previously [27,28]. ODT reconstructs the 3D RI tomogram of single-cell sample from multiple 2D images of the sample acquired with various illumination angles, which is similar to a computed tomography measuring X-ray absorptivity [29]. ODT measurements of single live cells were all performed at 37 °C in an atmosphere containing 5% CO_2_ using a commercial ODT microscope with excitation at 532 nm (HT-2H, Tomocube Inc. Daejeon, Korea) as previously described [27,28,29,30]. Briefly, a Mach–Zehnder interferometric microscope was used to reconstruct the cell 3D RI tomograms. The microscope comprises an illumination/sample modulation unit and an optical field recording unit. Optical interference is used to capture amplitude and phase information from light transiting a cell sample. The RI value of each medium was used as reference for ODT cell measurement. To verify the capability of the system, polystyrene beads with a diameter of 3 μm (Sigma-Aldrich Inc., St. Louis, MO, USA) or microspheres with a diameter of 6 μm (Polysciences Inc., Warrington, PA, USA) were used [30]. The power and exposure time of the illumination beam impinging on the sample were checked to verify that they did not negatively affect RI value measurements or image quality, and then fixed during ODT measurements. The lateral and axial optical resolutions of our ODT system were 200 nm and 1 μm, respectively. The resolution of RI values was <0.001 [29].

### 2.4. Image Rendering, RI, and Volume Calculation

The RI isosurfaces of the cytosol, chromosome, and plasma membrane of mitotic DM cells were rendered using TomoStudio software (2.6.25, Tomocube Inc., Daejeon, Korea) according to the manufacturer’s protocol and then compared with confocal images. The RI value was calculated using this software. Volume was calculated using Chromosome analysis software (1.1.7, Tomocube Inc., Korea). Briefly, user-defined transfer functions presenting a range of RI values and a range of the RI gradient magnitudes were changed using the virtual palette of TomoStudio until the 3D-rendered images of mitotic plasma membrane, cytosol, and chromosomes were sufficiently described compared with reference 3D images obtained using confocal microscopy (described in Section 3 and in the Supplementary Material). The 3D rendered images were compared with 3D images obtained using CLSM. The median value of the RI range was used for calculating mean RI values of the mitotic cytosol and chromosome under each medium condition.

### 2.5. Confocal Laser-Scanning Microscopy (CLSM)

Fluorescence microscopy of live cells was performed using an inverted confocal laser scanning microscope (LSM780; Carl Zeiss, Oberkochen, Germany). For 3D and time-lapse images, DM cells stably expressing H2B-mRFP and mGFP were illuminated at excitation wavelengths of 488 nm and 561 nm, and the emission signals were detected at 500–550 nm and 570–630 nm, respectively. Fixed DM cells stained with 4’,6-diamidino-2-phenylindole (DAPI) were illuminated at an excitation wavelength of 405 nm, and detected at 450–480 nm. HeLa cells transiently expressing H2B-mRFP were illuminated at an excitation wavelength of 561 nm, and the emission signal was detected at 570–630 nm. The interval for time-lapse imaging was 3 min, and z-stack images were acquired at 0.5-µm intervals using an objective lens (C-Apochromat, 63×, 1.2 NA). All live cell measurements were performed at 37 °C in an atmosphere containing 5% CO_2_. Fluorescence images were processed using Zen 2012 SP5 software (Carl Zeiss, Oberkochen, Germany of the LSM780 confocal microscopy system for 3D reconstruction or intensity profile analyses). Images acquired using the confocal microscope were 3D-rendered using IMARIS 8.1.2 software (Bitplane, Belfast, Ulster, USA) and used for volume analysis. The surface function of the IMARIS software was used required.

Cells were first cultured in a confocal microscope dish and fixed for 5 min with 1% paraformaldehyde and 1% glutaraldehyde in 0.1 M PBS and then stained for 5 min with DAPI. Images were enlarged five-fold using a 63× C-Apochromat lens, z-position thickness, in 0.50-µm units and 3D-rendered using Zen 2012 SP5 or IMARIS software. The 3D panoramic images were processed using IMARIS. All CLSM observations were performed at 25 °C.

### 2.6. Fluorescence Correlation Spectroscopy (FCS)

Confocal imaging for FCS measurements was performed using the LSM780 confocal microscope system. mGFP was excited at 488 nm using a CW Ar^+^ laser through a water-immersion objective (C-Apochromat, 40×, 1.2 NA; Carl Zeiss, Oberkochen, Germany). H2B-monomer RFP (mRFP) was imaged using a 561 nm diode pump solid state laser. To avoid bleed-through effects in double-scanning experiments, mGFP and mRFP were scanned independently in multitracking mode, and mRFP was scanned only for FCS measurements. All CLSM observations and corresponding FCS measurements were performed at 25 °C as previously described [3,31].

FCS measurements for detecting mGFP probe in DM cells were performed at 25 °C using an LSM780 confocal microscope (Carl Zeiss, Germany) as previously described [3,31]. mGFP was excited with minimized power at 488 nm with a continuous-wave Ar^+^ laser through a water-immersion objective (C-Apochromat, 40×, 1.2 NA; Carl Zeiss). H2B-mRFP was imaged using a continuous-wave 561 nm laser. Images were acquired using a GaAsP detector (Quasar; Carl Zeiss). Fluorescence autocorrelation functions (FAFs) were measured for 5 s at least three times at 2-s intervals to avoid non-stationary fluorescence fluctuations because of drift of the targeted chromosome during the measurements. To avoid bleed-through effects in double-scanning experiments, mGFP and mRFP were scanned independently using a multitracking mode, and only mRFP was scanned for FCS measurements. To confirm that the size of mitotic chromosome was sufficient to contain the confocal detection volume of FCS, 3D images of H2B-mRFP of mitotic DM cells were acquired before and after FCS measurements, as previously described [3].

Analysis of FCS data was performed as previously described [1,3]. To calculate the diffusion time, FAFs [G (*τ*)] of the measurements were fitted using the one-component model with or without a triplet term induced by a photochemical reaction as follows:

(1) G(τ)=1+1N(11+ττD)(11+(1s)2(ττD))12

where *N* is the number of molecules in the detection volume, *τ_D_* is the correlation time, *w* and *z* are the width and axial length of the detection volume, respectively, and *s* is the structure parameter *z*/*w*. Note that the triplet term is not shown for simplicity, because we only focus on the diffusional mobility, and the triplet is an independent term. Diffusion times as a function of diffusion coefficient were calculated as follows:

(2) $$\tau_{D}=\frac{w^{2}}{4D}$$

The diffusion of a spherical molecule is associated with various physical parameters by the Stokes–Einstein equation as follows:

(3) $$D=\;\frac{k_{B}T}{6\pi \eta r}$$

where *T* is the absolute temperature, *r* is the hydrodynamic radius of the spherical molecule, *η* is the fluidic viscosity of the aqueous solution, and *k_B_* is the Boltzmann constant. Because *D* is inversely proportional to viscosity, the viscosity of various solutions can be experimentally estimated using well-defined probe molecules such as mGFP [1,2,10]. The diffusion coefficient of mGFP (*D*_mGFP_) was calculated from the reported value of the diffusion coefficient of rhodamine 6G (*D*_Rh6G_ = 280 μm^2^/s), and the corresponding measured diffusion times of Rh6G (*τ*_Rh6G_) and mGFP (*τ*_mGFP_) were as follows [1]:

(4) $$\frac{D_{mGFP}}{D_{Rh6G}}=\frac{\tau_{Rh6G}}{\tau_{mGFP}}$$

All FAF curves for DM cell FCS measurements fitted well to a one-component model, using Zen 2012 SP5 software, as previously demonstrated [3]. Glycerol–water solutions containing Rh6G were measured, and the FAFs were analyzed to evaluate the *D* values and their corresponding viscosities [1].

### 2.7. Statistical Analysis

Student’s *t*-test or one-way analysis of variance (ANOVA) were performed to evaluate the significance of differences in mean values (Origin v.8.5; Northampton, MA, USA), and *p* < 0.01 were considered significant.

## 3. Results and Discussion

### 3.1. Comparisons of Images of Chromosomes Acquired Using ODT and 3D CLSM

**Figure 1:**
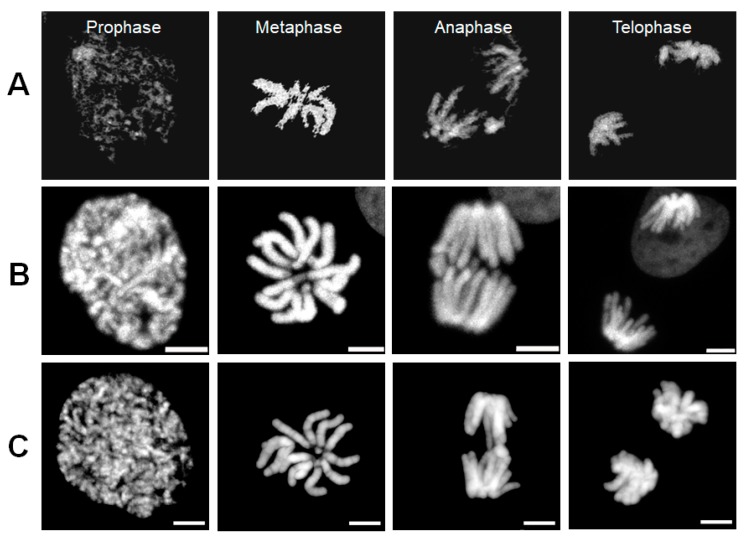
Comparison of 3D confocal fluorescence images of the H2B marker protein tagged with monomeric RFP (H2B-mRFP) with 3D RI images of chromosomes during mitosis. (**A**) Four RI images of a DM cell stably expressing H2B-mRFP and monomeric green fluorescent protein (mGFP) from a series of time-lapse observation during mitosis. For clarity, only high and continuous RI regions of chromosome in mitotic cytosol are shown (see also Supplementary Movie 1). (**B**) Four fluorescence images of H2B-mRFP from a series of time-lapse and 3D observations depicting mitosis in a DM cell stably expressing H2B-mRFP and mGFP. For clarity, images from mGFP channels are not shown. (**C**) Four fluorescence images of mitotic chromosomes of fixed DM cells stained by DAPI. Scale bar = 5 μm.

To demonstrate how RI images of mitotic chromosomes were accurately constructed through ODT measurements, live DM cells expressing H2B-mRFP or mGFP were measured using ODT. Because of the small number and large size of the DM chromosomes, we expected that tomographic RI images of DM cells may show a clear chromosomal structure representative of the characteristic properties of chromosomes during mitosis. For each ODT and CLSM measurement, label-free and 3D RI images of the mitotic chromosomes of a DM cell acquired from time-lapse ODT measurements and analyses were compared with fluorescent 3D images of H2B-mRFP acquired during mitosis of other DM cells using by confocal microscopy (Figure 1, Movie S1, S2). Figure 1A shows four tomographic RI images of a DM cell chromosome from prophase to telophase, and Figure 1B shows the corresponding representative fluorescence images from live DM cells expressing H2B-mRFP. Figure 1C shows the corresponding representative fluorescence images from fixed DM cells stained using DAPI. Note that the sharp and rough surfaces of chromosomes in ODT images are characteristic compared with the round and even surfaces of the CLSM 3D images of chromosomes. In principle, the quality of absolute RI values and ODT images is not altered by the excitation power and exposure time used in this study. It is likely that chromosomal surfaces with RI similar to or lower than the surrounding cytosolic organelles are not clearly differentiated by the image-rendering process. The reason for the difference in the apparent surfaces between label-free and fluorescent images remains unclear.

Using time-lapse and 3D confocal imaging (Figure 1B, Figure S1a, Movie S3), there were seven diploid chromosomes, which is consistent with the number of chromatids in male DM cells. In contrast, it was difficult to image and count the number of chromatids in live HeLa cells clearly using time-lapse and 3D CLSM and ODT images, respectively (Figure S1b, Movie S4, S5). RI images of DM cell chromosomes in prophase were unclear compared with those of other mitotic phases and compared with the fluorescence images obtained using CLSM because of heterogeneous and punctate structures of discontinuous and differing RI values. The unclear structural features of prophase chromosomes such as thickness and boundaries was significantly changed when small differences in user-defined function were used. Therefore, we selected the RI image of chromosomes in prophase that was similar to that of representative confocal images. In contrast, RI images of chromosomes in metaphase, anaphase, and telophase were clearly homogenous and showed the long arm of the thick chromatid. No clear chromosomal structure in prophase HeLa cells was found using ODT, even though the RI images of chromosomes in other phases were clearly reconstructed (Movie S4). However, the arm of the chromatid of mitotic HeLa cells could not be imaged, and chromosomes resembled rod-like lumps. The number of diploid chromosomes detected using CLSM could not be precisely determined from the RI images of metaphase or anaphase cells. Thus, our present method has a limitation for exact description of chromosome at each phase because of measurements conducted on two different instruments of ODT and CLSM. Nevertheless, the images showed that ODT measurements allowed successful reconstruction of dynamic structural changes in the mitotic chromosomes of DM cells from metaphase to telophase. Combined and correlative methods of label-free and fluorescence in a single instrument will be facilitate such comparisons as previously demonstrated [26,32].

### 3.2. Quantification of the RI Values of Mitotic Chromosomes as a Function of Osmotic Conditions

**Figure 2:**
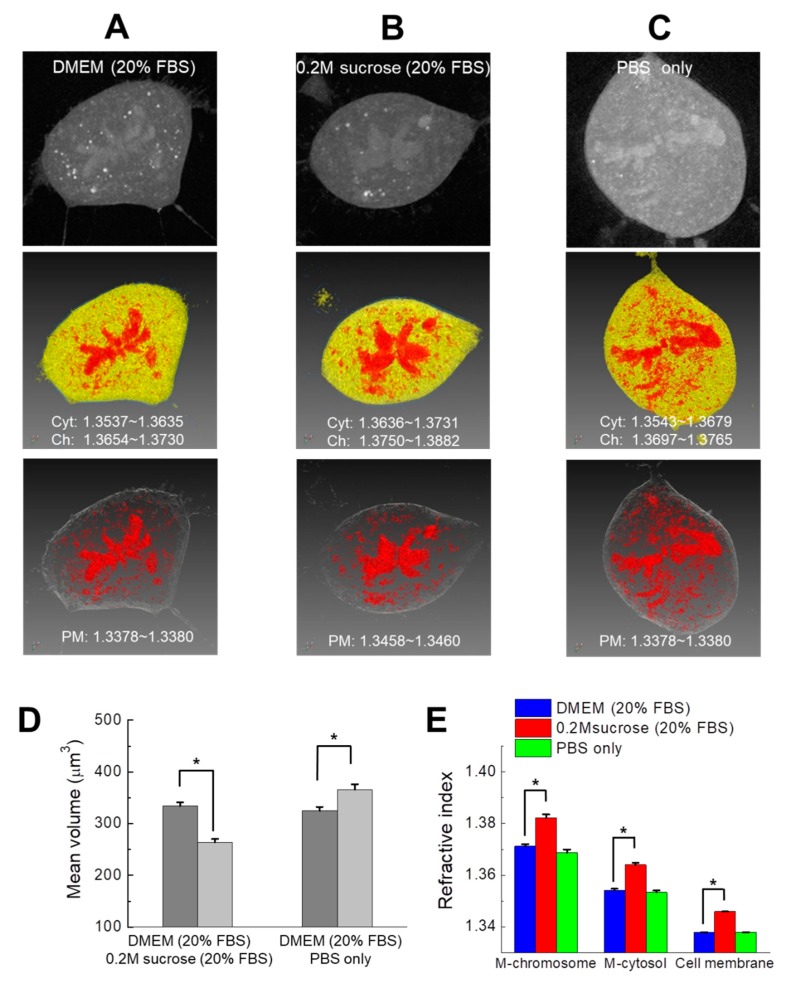
Detection of RI changes in chromosomes of DM cells under different osmotic conditions. (**A**–**C**) Representative raw optical diffraction tomography (ODT) 3D images (upper), pseudocolored RI 3D images (center) of cytosol (yellow), chromosomes (red), and plasma membranes (blue) during metaphase of live DM cells under normal (DMEM), hypertonic (0.2 M sucrose), and hypotonic (PBS) conditions respectively. For clarity, pseudocolored RI images of plasma membranes (white) and chromosomes (red) are shown (bottom). The pseudocolor images of mitotic cytosol and chromosomes are represented by yellow and red, respectively. Corresponding RI ranges of mitotic cytosol (Cyt) and chromosomes (Ch) are indicated (inset). (**D**) The mean change of volumes change of all chromosomes of one mitotic cell after media exchange was evaluated using 3D confocal imaging of individual DM cells expressing H2B-mRFP (mean ± standard error of the mean (SEM); n = 25 cells). Identical single DM cells were respectively traced and imaged before (gray) and after (light gray) media exchange as indicated. * *p* < 0.01. (**E**) Mean values of RI of mitotic chromosomes in metaphase, cytosol, and membranes under each condition (mean ± SEM; n = 20 cells). The RI value of the cell membrane was mostly independent of the media conditions. M denotes mitotic cells. * *p* < 0.01 for M-chromosome and M-cytosol and * *p* < 10^−6^ for cell membrane.

Confocal and electron microscopy revealed that nuclear chromatin is highly compact and reorganized in hypertonic media condition [6]. Therefore, we expected that the volume and RI (i.e., molecular density) of chromosomes in mitotic cells may change according to osmolarity. To investigate the influence of osmotic changes on the structure and physicochemical properties of mitotic chromosomes in live DM cells, the physiological state of cells was changed using culture media of varying osmotic pressure (Table 1 and Section 2) [6]. Live DM cells are normally cultured under relatively high osmotic pressure in DMEM supplemented with 20% FBS, and hypertonic conditions were achieved by adding 0.2 M sucrose. In contrast, relatively hypotonic conditions were achieved by substituting PBS for the media, although the osmotic pressure of PBS is lower compared with that of DMEM media. RI value of mitotic chromosomes, cytosol, and plasma membrane was measured by ODT (Figure 2A–C, Figure S2a–c).

The volumes of mitotic DM cell chromosomes were evaluated using ODT (Figure S2d). Figure 2A–C shows representative raw ODT and pseudocolored RI images of live DM cells in metaphase cultured in three different osmotic conditions. Note that the same pseudocolor of the cytosol, chromosome, and membrane was used for structural clarity and comparison between conditions. Instead, the ranges of RI values of the mitotic cytosol and chromosomes in each medium are indicated. Mitotic chromosomes appeared as large and continuous rod-like RI regions at the center of the mitotic cell. However, many small and dot-like unknown structures were detected throughout the cytosol. Such small cytosolic bodies were not detected in the confocal observations of the mitotic chromosomes in DM cells expressing H2B-mRFP (Figure 1B). Therefore, these structures likely are not components of the chromosome, but may be other cytosolic organelles or subcellular bodies such as vesicles and mitochondria. For this reason, we excluded such small organelles with similar RI values to the chromosome region from evaluation of chromosome volume before comparison (Figure 1A, Figure 2A, Movie S6).

Although the mean value of the chromosome volume of cells acquired from ODT varied with osmotic conditions, significant changes in volumes were only detected between hypertonic and hypotonic conditions (Figure S2d). It is likely that excluding small organelles from cytosol affected the accuracy of the volume evaluation. Moreover, it will be helpful for investigating changes in chromosome volumes with identical single-cell varying conditions. Accordingly, CLSM observations of identical single DM cells expressing H2B-mRFP was separately performed for the evaluation of the chromosome volume because of the limitation of the ODT instrument for observations of identical single cells. Interestingly, 3D confocal image analysis of identical single DM cells before and after media change indicated that the mean mitotic chromosome volume was significantly decreased or increased by hypertonic and hypotonic culture conditions, respectively (Figure 2D, Figure S3). However, the values of the chromosome volumes obtained from ODT and CLSM were significantly different. Although the reason for this discrepancy between ODT and CLSM analyses is unclear, parameters such as excessive elimination of small bodies in ODT images or overestimation of fluorescence signals in CLSM observations may contribute to the discrepancy.

The mean RI values of mitotic cytosol, chromosomes, and plasma membranes under each culture condition are summarized in Figure 2E and Table 2. The mean RI values of the mitotic cytosol and chromosomes in normal DMEM were significantly increased by strong hypertonic treatment, while under weak hypotonic conditions, RI values did not significantly decrease compared with those determined under normal culture conditions. Physically, it is expected that a decrease of chromosomal volume induces an increase of molecular density and vice versa. Because of a small difference (50 mOsm/kg) of osmolarity between DMEM and PBS culture, it is likely that RI is not changed enough to be differentiated in such conditions. Otherwise, RI and chromosomal volume detections from ODT have limited sensitivity to such small differences of physiological state (Figure S2D), while CLSM evaluation can detect the small change of volume (Figure 2D). Nevertheless, the results suggest that a large change of osmotic condition (over 50 mOsm/kg) induces reorganization of molecule density in mitotic chromosomes and can be detected by ODT analyses. The mean RI value of the cell membrane hardly varied among cells under the same culture conditions. Although the RI value of the plasma membrane under hypertonic conditions is significantly higher compared with those of normal and hypotonic culture conditions, it is noteworthy that the high RI value of the membrane can be overestimated because of an increase of RI in the culture media and the cytosol, because the plasma membrane is much thinner than the spatial resolution of ODT. For example, previous studies found that osmotic changes or molecular crowding induces reorganization of the nuclear structure, as well as a noticeable compaction of chromatin in the interphase nucleus [6,12,33,34,35]. To confirm chromatin compaction in the interphase nucleus, the same individual DM cells expressing H2B-mRFP were analyzed before and after hypertonic or hypotonic treatment (Figure S4). Fluorescent chromatin in the nuclei of individual DM cells was observed using confocal microscopy before and after treatment. Chromatin was significantly compacted following hypertonic treatment (Figure S4a,c,e,g,i). In contrast, the distribution of chromatin did not significantly change under hypotonic treatment (Figure S4b,d,f,h,j). These findings are in agreement with previous confocal and electron microscopy observations [6,7]. The molecular components of chromatin are similar to those of the chromosome, suggesting that compaction of mitotic chromosomes may be induced by hyperosmotic conditions and may be accompanied by increased molecular density, if there is no loss of molecular components in the chromosome under hypertonic conditions [36]. The results of our ODT analysis is consistent with this possibility.

### 3.3. Quantification of the Diffusional Mobility of Monomeric Green Fluorescent Protein (mGFP) in Mitotic Chromosomes under Different Osmotic Conditions

**Figure 3:**
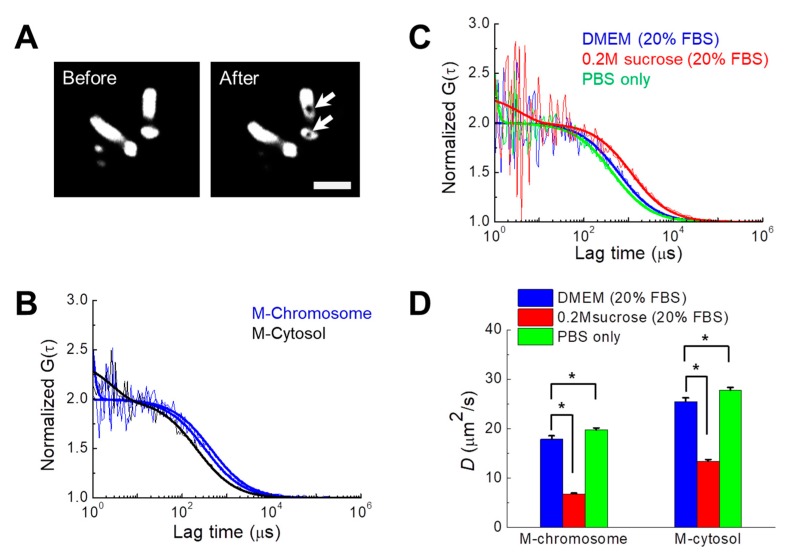
Changes in *D* values of mGFP probes in chromosomes of DM cells under different osmotic conditions. (**A**) Images of mitotic chromosomes (H2B-mRFP) during metaphase of a DM cell before and after two positions of fluorescence correlation spectroscopy (FCS) measurements. The positions of FCS measurements in the chromosome are clearly bleached (arrow), verifying the actual measured region. Scale bar = 5 μm. (**B**) FCS analysis of mGFP expressed in a DM cell during mitosis under normal conditions. Fluorescence autocorrelation function (FAF) curves measured in a position of the mitotic cytosol and two positions of mitotic chromosome are shown. (**C**) FAF curves of mitotic chromosomes under three different osmotic conditions. For comparison of mobilities, all curves in (**B**) and (**C**) were normalized to the same amplitude, *G* (0) = 2. Solid lines indicate fitting of a one-component free diffusion model to the results. (**D**) Mean *D* values of mGFP in mitotic chromosomes and the cytosol during mitosis under the three different conditions are shown (mean ± SEM; n = 20 cells). * *p* < 0.01.

Tracking the mobility (i.e., the diffusion coefficient, *D*) of probe molecules is a well-known method for calculating the local viscosity of fluids and molecular crowding in cells and aqueous solutions [1,3,37]. As previously demonstrated [3], diffusional mobility of mGFP was quantified in the cytosol and chromosomes during mitosis in live DM cells under varying osmotic conditions (Figure 3). The volumes of mitotic chromosome were larger in DM cells compared with those of other cell lines (Figure 3A). Therefore, it was feasible to target and measure the FCS of mitotic chromosomes and the cytosol of single DM cells as previously demonstrated [3]. Consistent with previous findings [3], the fluorescence autocorrelation curves of DM cells fit well to the single component model of free diffusion with or without a triplet term, revealing that the diffusional mobility of mGFP in mitotic chromosomes was much lower compared with that in mitotic cytosol (Figure 3B). Moreover, the sharp curves at the region of short lag times under 10 μs corresponds to the triplet term generated by the photochemical process.

The diffusional mobility of mGFP in the cytosol and chromosomes significantly changed as a function of osmotic pressure (Figure 3C,D). Table 3 summarizes the mean *D* values of mGFP in the mitotic cytosol and chromosomes under each medium condition. The average *D* values of mGFP in the mitotic cytosol and chromosomes significantly decreased under hypertonic culture conditions. Although the relative change (<10%) induced by hypotonic conditions was smaller compared with that induced by hypertonic conditions (>40%), the *D* values were significantly increased under the former condition. These results demonstrate that the mobility of the probe is much more sensitive to changes in the cellular microenvironment induced by osmotic stress. Nevertheless, the FCS results are consistent with the results showing that the RI of chromosomes was influenced by osmotic stress (Figure 2E).

### 3.4. Relationship between Chromosomal RI and Diffusion of Intrachromosomal mGFP

**Figure 4:**
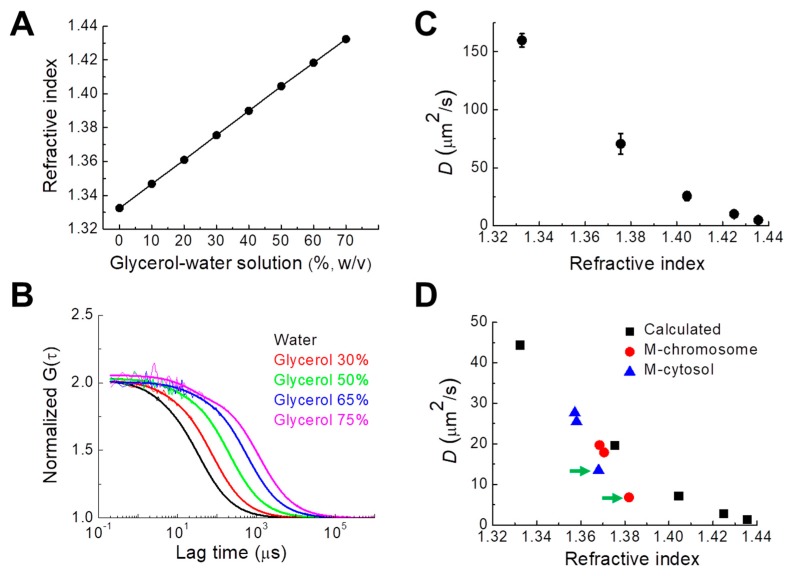
Experimental determination of the relationship between the *D* values of rhodamine 6G and the RIs of the medium. (**A**) Measured RI values of water and various glycerol–water solutions of known concentration at 25 °C. Note that the standard error was smaller than 0.00005 and is not shown in the plot. (**B**) Measured FAF curves of rhodamine 6G in water and four different glycerol–water solutions of known concentration. For comparison of mobilities, all functions were normalized to the same amplitude, *G* (0) = 2. Bold solid lines indicate fitting of one-component diffusion model to the results. The fitting results were used for evaluating *D* value. (**C**) Relationship between the RI values of the solutions obtained from **A** and the *D* values of rhodamine 6G in glycerol–water solutions obtained from **B**. (**D**) Plot of the calculated *D* values of mGFP in glycerol–water solutions with known viscosity vs. RI values (black) measured at 25 °C. Measured *D* values of mGFP in mitotic chromosomes (red) and cytosol (blue) of DM cell under hypotonic, DMEM, and hypertonic culture conditions are shown. The RI values of mitotic cytosol and chromosomes measured at 37 °C were compared with the values at 25 °C, according to the linear relation between RI and density values at two different temperatures (Table 1, Figure S5).

To quantify the relationship between the RI of the aqueous medium and the diffusion coefficient of fluorescent probes molecules in the medium (i.e., viscosity of the medium), we measured the RIs of water and glycerol–water solutions of different densities and evaluated the *D* values of rhodamine 6G in the solutions. There were linear relations between the RI and the molecular density of glycerol solution at 25 °C and 37 °C, respectively (Figure 4A, Figure S5). The result is in good agreement with the known properties of glycerol–water solution [37]. Previous studies found that the cellular viscosity of cultured cells is much higher compared with that of pure water [1,3,38]. The viscosity of solutions with glycerol concentrations ranging from 20% to 50% (*w*/*w*) is similar to the fluidic viscosity of intracellular compartments. Since the viscosities of glycerol–water solutions are also well known [37], *D* values of rhodamine 6G (0.479 kD) in glycerol–water solutions were experimentally determined from FCS measurements (Figure 4B, see also Methods). Comparison of measured RI and *D* values establishes the relationship between RI and *D* (Figure 4C). From the model experimental results and the Stokes–Einstein relationship, theoretical *D* values of mGFP (molecular mass 28 kDa) in glycerol–water solutions were calculated [1]. The mean *D* values (Figure 3D, Table 3) of mGFP measured in the mitotic cytosol (blue) and DM chromosomes (red) were plotted for comparison with the theoretical *D* values (Figure 4D). For DMEM and PBS media, the measured and theoretical *D* values of mGFP in mitotic DM cells were consistent. In contrast, the *D* value of DM cells cultured in hypertonic media was significantly smaller compared with the theoretical value. Hypertonic stress enforces molecular crowding in cellular components because of the osmotic extraction of water from the cells [6], suggesting that molecular diffusion in the cytosol and chromosome under conditions of high osmolality is largely affected by molecular crowding induced by hypertonic conditions [4,39]. Otherwise, such water extraction can restrict nucleosome movement in the chromosome, which significantly slows the diffusion of probes [3]. Moreover, these large changes of RI and *D* values of mitotic chromosomes of DM cells are similar to those for the interphase cytosol and nucleoplasm, but different from that of the interphase nucleolus as previously demonstrated [28].

## 4. Conclusions

Here we demonstrate a new method to examine the physicochemical properties of the mitotic chromosomes of live cells by combining label-free ODT imaging with complementary confocal microscopy and FCS analyses. Our experimental approach assumed that the RI acquired from ODT analysis is quantitatively related to the diffusion coefficient of the probes used for FCS analysis. We verified 3D RI values of images of mitotic chromosomes in DM cells expressing H2B-mRFP and mGFP through a comparison with 3D confocal images. Finally, we examined the effects of osmotic stress on the RIs of mitotic cells and the diffusion coefficient of the mGFP probe. Moreover, we demonstrated an inverse relationship between the RI and diffusion coefficient in live cells during mitosis. Interestingly, we found that the chromosomes were accessible to probe molecules that freely diffuse, although hypertonic stress significantly increased the molecular density of the chromosome. Our results confirm and extend those of previous studies concerning the influence of osmotic stress on nuclear structure and molecular crowding effects. Through the new complementary pairing of ODT, confocal, and FCS analysis, further insights are provided into the physicochemical properties of chromosomes and other subcellular compartments with a high macromolecular content in live cells.

## Figures and Tables

**Table 1:** Measured osmolarity and refractive indices of culture media and solutions at 25 °C and 37 °C.

	DMEM ^a^ (20% FBS)	0.2 M Sucrose ^b^ (20% FBS)	DMEM ^c^ (10% FBS)	0.2M Sucrose (10% FBS)	PBS only	Distilled Water (Reference)
Osmolarity (mOsm/kg)	~340	~500	~330	~500	~290	-
RI (25 °C)	1.3365	1.3456	1.3358	1.3447	1.3349	1.3325
RI (37 °C)	1.3350	1.3441	1.3342	1.3431	1.3336	1.3310

^a^ Dulbecco’s modified Eagle’s medium (DMEM) containing 20% fetal bovine serum (FBS) and ^b^ DMEM containing 20% FBS and 0.2 M sucrose were used as media for Indian Muntjac (DM) cells. ^c^ DMEM containing 10% FBS was used for HeLa cells. Dulbecco’s phosphate-buffered saline (PBS) was used for DM and HeLa cells. The refractive index (RI) of distilled water is shown as a reference. RI was measured at 488 nm.

**Table 2:** Summary of refractive indices of mitotic chromosomes and cytosol in live DM cells cultured at 37 °C.

Media Condition	M-Chromosomes	M-Cytosol	Cell Membrane (Control) ^d^
DMEM (20% FBS) ^a^	1.3712 ± 0.0009	1.3541 ± 0.0006	1.3379
0.2 M sucrose ^b^ (20% FBS)	1.3822 ± 0.0013	1.3640 ± 0.0008	1.3459
PBS ^c^ only	1.3688 ± 0.0012	1.3534 ± 0.0007	1.3379

Refractive indices (RIs) of mitotic chromosomes in live DM cells cultured at 37 °C were evaluated using software installed on an ODT instrument (mean ± SEM; n = 20). ^a^ DMEM for DM cell culture contains 20% FBS and ^b^ hypertonic DMEM medium contains 20% FBS and 0.2 M sucrose. ^c^ Dulbecco’s PBS was used as hypotonic media (see Section 2). ^d^ RIs of the cell membranes did not significantly change as a function of osmotic state and are shown as a reference.

**Table 3:** Summary of diffusion coefficients (*D*) of mGFP in mitotic chromosomes and cytosol of live DM cells.

Media Condition	M-Chromosome (μm^2^/s)	M-Cytosol (μm^2^/s)
DMEM (20% FBS)	17.9 ± 0.7	25.5 ± 0.8
0.2 M sucrose (20% FBS)	6.8 ± 0.2	13.5 ± 0.3
PBS only	19.7 ± 0.4	27.7 ± 0.6

Diffusion coefficient (*D*) was calculated through fitting to fluorescence correlation spectroscopy data (mean ± SEM; n = 20).

## Supplementary Materials

The following are available online at https://www.mdpi.com/2073-4409/8/11/1368/s1, Figure S1: The number of chromatids of a DM cell can be clearly imaged and counted, Figure S2: Influence of osmotic changes on the mean RI and volume of mitotic chromosomes in live DM cells, Figure S3: Structural change of mitotic chromosome observed by 3D reconstruction of confocal images of single DM cells coexpressing H2B-mRFP and mGFP, Figure S4: Influence of osmotic change on nuclear distribution of chromatin of DM cells. Nuclear reorganization by osmotic change was analyzed by using confocal images of single DM cells coexpressing H2B-mRFP and mGFP, Figure S5: Linear relationship between refractive index and viscosity of glycerol–water solution.
