# Supplementary material for: Mitotic Chromosomes in Live Cells Characterized Using High-Speed and Label-Free Optical Diffraction Tomography
Source: Cells. 2019 Oct 31;8(11):1368. doi: 10.3390/cells8111368 (PMC6912651; doi:10.3390/cells8111368)
Supplement: Supplementary file 1 [file cells-08-01368-s001.zip › cells-608548-supplementary-for XML/cells-608548-supplementary-for XML.docx]

**Supplementary data**

**Mitotic Chromosomes in Live Cells Characterized Using High-Speed and Label-Free Optical Diffraction Tomography**

**Tae-Keun Kim ^1,†^, Byong-Wook Lee ^1,†^, Fumihiko Fujii ^2^, Kee-Hang Lee ^3^, Sanghwa Lee ^1^, YongKeun Park ^4^, Jun Ki Kim ^1,5,^*, Sang-Wook Lee ^6,^*, Chan-Gi Pack ^1,5,^***

^1^ Asan Institute for Life Sciences, Asan Medical Center, Seoul 05505, Republic of Korea

^2^ Division of Physical Pharmacy, Faculty of Pharmaceutical Sciences, Kobe Gakuin University, Kobe 650-8586, Japan

^3^ TOMOCUBE Inc., Daejeon 34051, Republic of Korea

^4^ Department of Physics, Korea Advanced Institute of Science and Technology, Daejeon 34141, Republic of Korea

^5^ Department of Convergence Medicine, University of Ulsan College of Medicine, Seoul 05505, Republic of Korea

^6^ Department of Radiation Oncology, University of Ulsan College of Medicine, Asan Medical Center, Seoul 05505, Republic of Korea

***** Correspondence: [kim@amc.seoul.kr](mailto:kim@amc.seoul.kr) (J.K.K.), [lsw@amc.seoul.kr](mailto:lsw@amc.seoul.kr) (S.-W.L.), [changipack@amc.seoul.kr](mailto:changipack@amc.seoul.kr) (C.-G.P.)

**^†^** These authors contributed equally to this work.


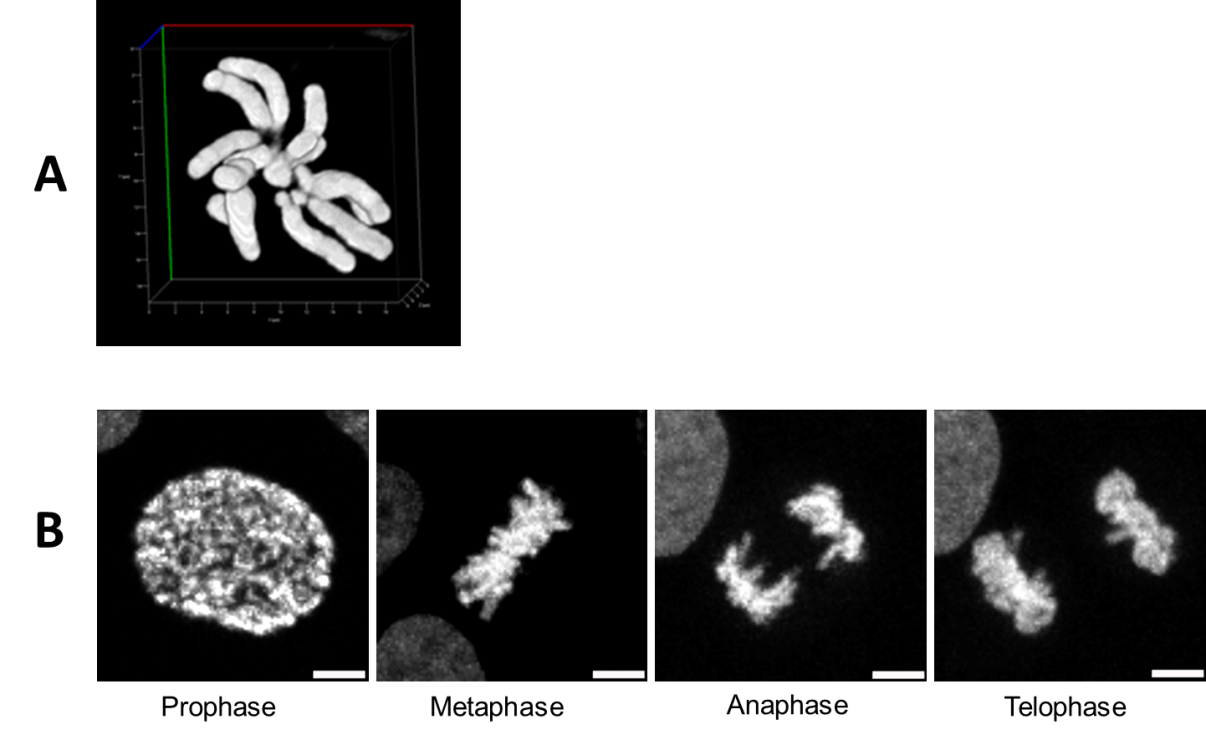


**Figure S1.** The number of chromatids of a DM cell can be clearly imaged and counted. (**A**) The number of chromosomes in a fixed DM cell was counted from z-stacked 3D images of H2B-mRFP (see also Supplementary Movie S3). (**B**) 3D and time-lapse fluorescence image of H2B-mRFP expressed in a live HeLa cell. Four fluorescence images of H2B-mRFP are shown from a series of time-lapse observations depicting mitosis in a HeLa cell transiently expressing H2B-mRFP. Scale bar represents 5 μm.


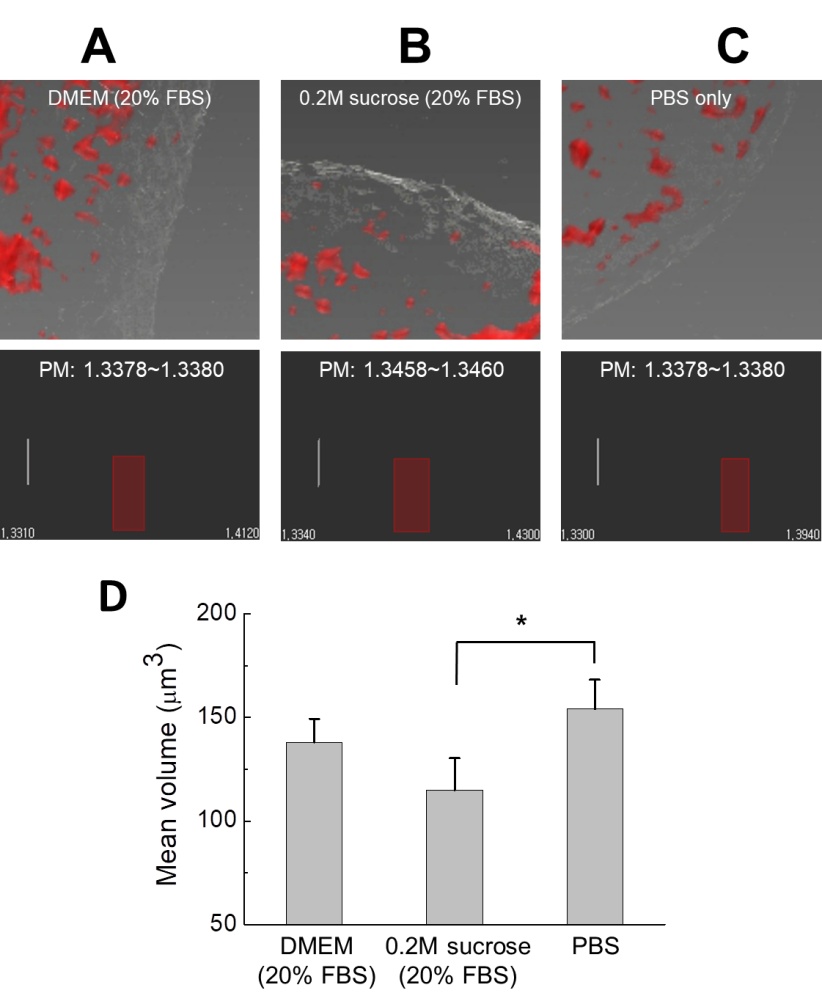


**Figure S2.** Influence of osmotic changes on the mean RI and volume of mitotic chromosomes in live DM cells. (**A–C**) Raw ODT images, pseudo-colored RI images of chromosomes (Ch, red), and plasma membrane (PM, light gray) of mitotic cells, and corresponding virtual palettes for RI range are shown. The ranges of each RI value for membrane and chromosomes of the mitotic cells are also shown. Median values of the limited RI ranges are used for calculation of RI values of membrane and chromosomes of live cells. (**D**) Mean volume of all chromosomes in one mitotic cell at the three different osmotic conditions (means ± SEM; n=20 cells) obtained by ODT measurement of DM cells. Physiological (DMEM with 20% FBS), hypertonic (0.2 M sucrose with 20% FBS), and relatively hypotonic (PBS) cell culture media were used for osmotic change. * p > 0.01.


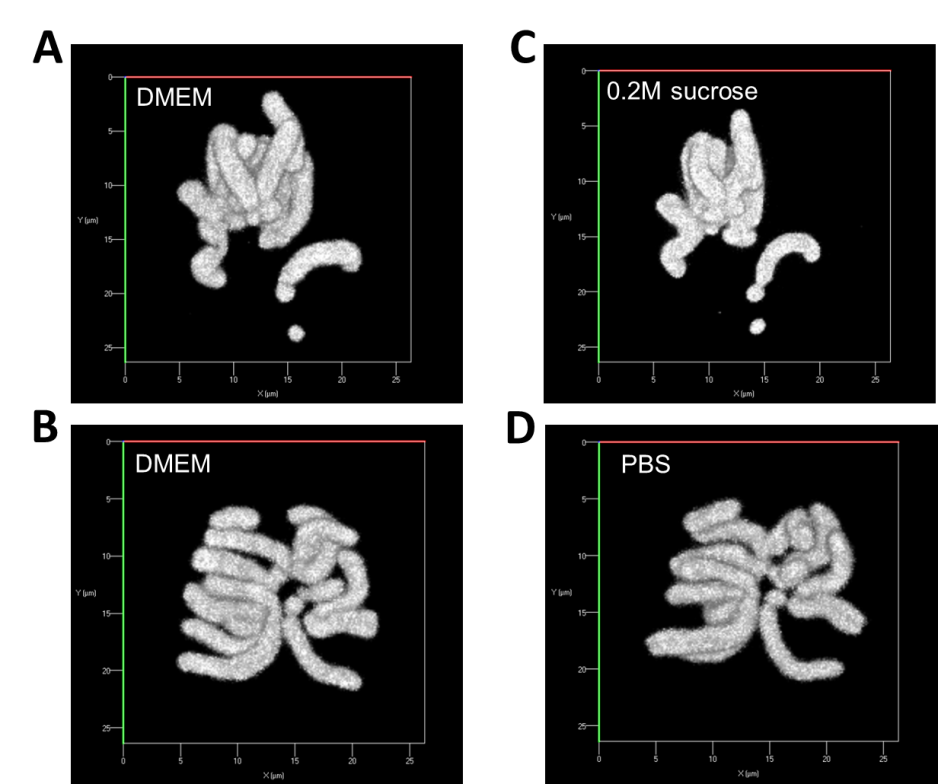


**Figure S3.** Structural change of mitotic chromosome observed by 3D reconstruction of confocal images of single DM cells coexpressing H2B-mRFP and mGFP. (A, C) Mitotic chromosomes of a DM cell in metaphase before and after treatment of 0.2 M sucrose media are shown. (B, D) Mitotic chromosomes of a DM cell before and after treatment of PBS media are shown.


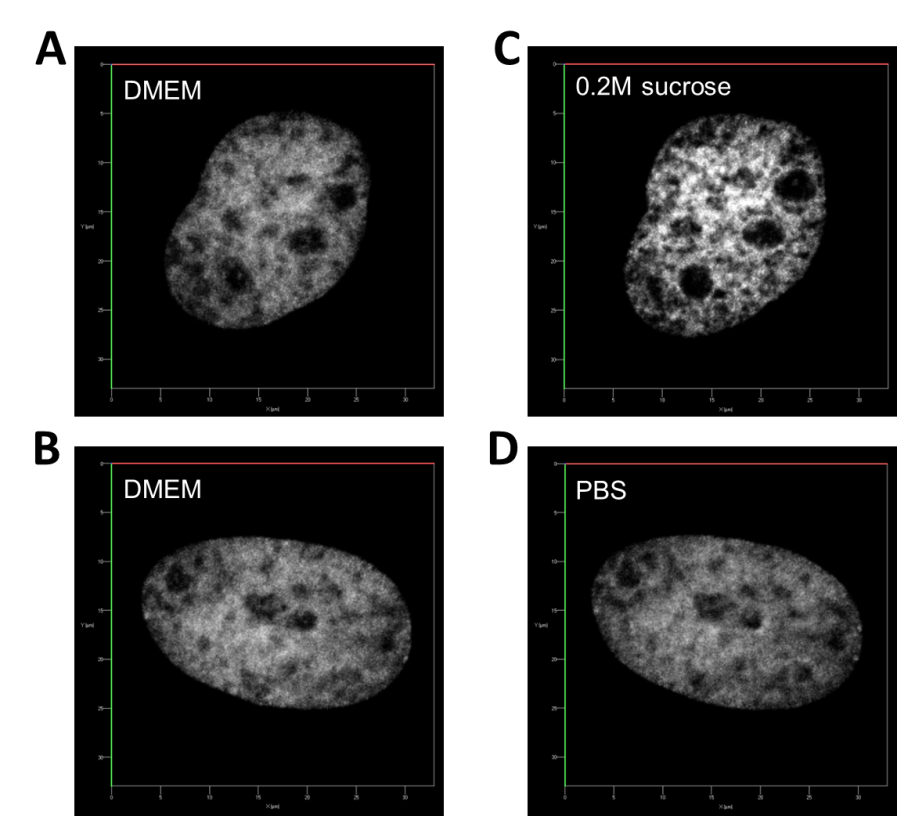


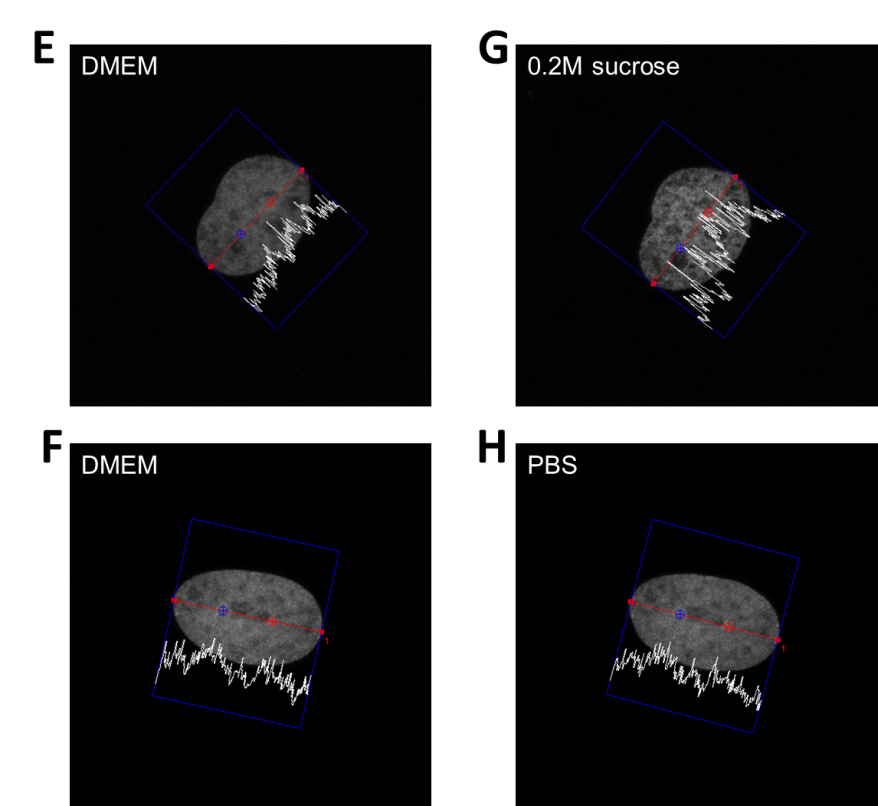


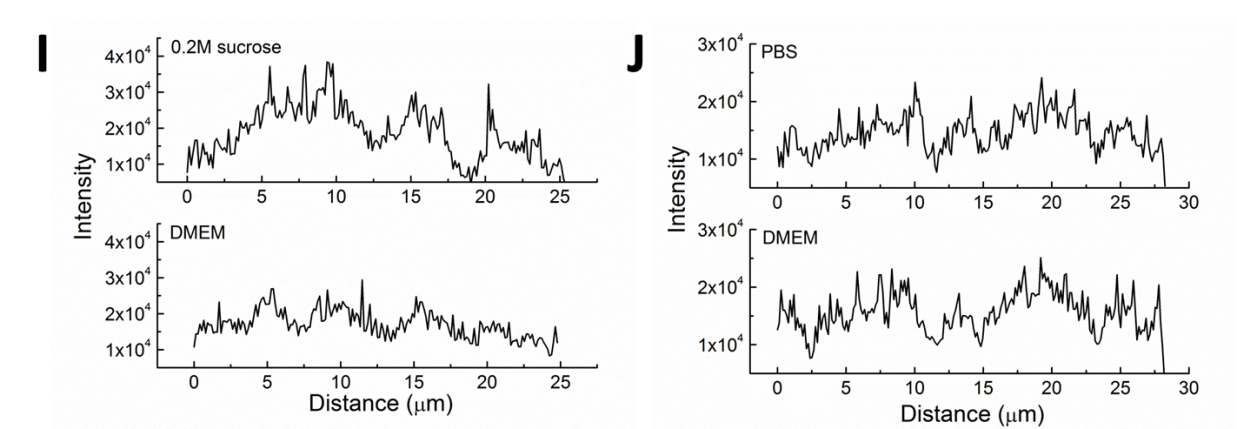


**Figure S4.** Influence of osmotic change on nuclear distribution of chromatin of DM cells. Nuclear reorganization by osmotic change was analyzed by using confocal images of single DM cells coexpressing H2B-mRFP and mGFP. (**A, C**) Nuclear re-distribution of H2B-mRFP expressed in a DM cell before and after treatment of 0.2 M sucrose media is shown. (**B, D**) Nuclear distribution of H2B-mRFP expressed in a DM cell before and after treatment of PBS media is shown. (**E, G**) Linear profiles of fluorescent intensity of H2B-mRFP along the longitudinal axis in the nucleus of a DM cell shown in (**A, C**) are indicated. (**F, H**) Linear profiles of fluorescent intensity of H2B-mRFP along the longitudinal axis in the nucleus of a DM cell shown in (**B, D**) are indicated. (**I**) Comparison of fluorescent intensity profile of chromatin in DMEM media (lower) and after treatment of 0.2 M sucrose media (upper). (**J**) Comparison of fluorescent intensity profile of chromatin in DMEM media (lower) and after treatment of PBS media (upper).


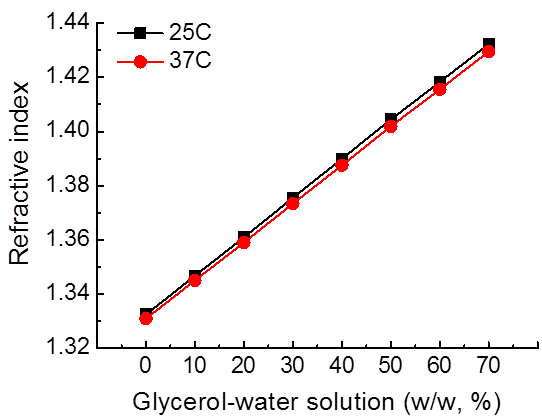


**Figure S5.** Linear relationship between refractive index and viscosity of glycerol–water solution. Refractive index of glycerol–water solution with known glycerol concentration was measured at 25 °C and 37 °C, respectively.
